# The effect of hyperbaric oxygen therapy on the pathophysiology of skin aging: a prospective clinical trial

**DOI:** 10.18632/aging.203701

**Published:** 2021-11-16

**Authors:** Yafit Hachmo, Amir Hadanny, Sonia Mendelovic, Pnina Hillman, Eyal Shapira, Geva Landau, Hadar Gattegno, Avi Zrachya, Malka Daniel-Kotovsky, Merav Catalogna, Gregory Fishlev, Erez Lang, Nir Polak, Keren Doenyas, Mony Friedman, Yonatan Zemel, Yair Bechor, Shai Efrati

**Affiliations:** 1 Research and Development Unit, Shamir Medical Center, Zerifin, Israel; 2 The Sagol Center for Hyperbaric Medicine and Research, Shamir (Assaf-Harofeh) Medical Center, Zerifin, Israel; 3 Sackler School of Medicine, Tel-Aviv University, Tel-Aviv, Israel; 4 Bar Ilan University, Ramat-Gan, Israel; 5 Pathology Department, Shamir (Assaf-Harofeh) Medical Center, Zerifin, Israel; 6 Plastic Surgery Department, Shamir (Assaf-Harofeh) Medical Center, Zerifin, Israel; 7 Sagol School of Neuroscience, Tel-Aviv University, Tel-Aviv, Israel

**Keywords:** aging, skin, hyperbaric oxygen, senescence, angiogenesis

## Abstract

Introduction: Skin biopsies can be used to evaluate physiological effects of aging targeted intervention at the tissue/cellular levels. Recent clinical trials have shown that hyperbaric oxygen therapy (HBOT) can target aging hallmarks, including telomere shortening, senescent cells clearance and angiogenesis. The aim of this study was to evaluate the effects of HBOT on the skin of a normal, non-pathological, aging population.

Methods: The study was performed as a prospective clinical trial. After signing informed consent and undergoing baseline evaluations, the subjects were assigned to a three-month control period followed by three months of HBOT daily sessions. Skin biopsies were taken at baseline, after three months of no intervention (control) and 1-2 weeks following the last HBOT session. Trichrome, Orecin, lipofuscin and CD31 staining were used to evaluate collagen fibers, elastic fibers, senescent cells and blood vessels, respectively.

Results: Out of the cohort of 70 participants in the normal aging population study, thirteen male patients (age 68.07±2.5y) gave consent for repeated skin biopsies. Following HBOT, there was a significant increase in collagen density (p<0.001, effect size(es)=1.10), elastic fiber length (p<0.0001, es=2.71) and the number of blood vessels (p=0.02, es=1.00). There was a significant decrease in fiber fragmentation (p=0.012) and in tissue senescent cells (p=0.03, es=0.84) post-HBOT. No changes were noted in elastic fiber density or thickness.

Conclusions: The study indicates, for the first time in humans, that HBOT can significantly modulate the pathophysiology of the skin aging in a healthy aging population. The demonstrated mechanisms include angiogenesis and senescent cell clearance.

## INTRODUCTION

Aging can be characterized by the progressive loss of physiological integrity, culminating in impaired function and susceptibility for diseases and death. Being the most voluminous organ of the body, the skin is no exception. There are two types of skin aging, intrinsic and extrinsic aging. Intrinsic aging, also referred as chronological aging, includes the pathophysiological processes common to most organs in our body, while extrinsic aging or photoaging, is unique to the skin and is related to the effects of ultraviolet (UV) radiation (mainly originating from the sun) and other environmental agents that the skin is exposed to.

Intrinsic aging, is related to well-known cellular aging hallmarks including cell senescence, telomere shortening, genomic instability, inflammation and mitochondrial dysfunction [1–3]. Another relevant factor is the weakening of extracellular matrix molecules, such as collagen and elastin. The most remarkable histological changes occur within the basal cell layer: proliferation of keratinocytes, fibroblasts, and melanocytes cells in the basal layer reduces the epidermis which becomes thinner, resulting in a decrease of the contact surface area between the dermis and epidermis layers [4]. In addition, the width of the papillary dermis increases with reduced collagen fibers, elastic fibers, mast cells and fibroblasts [5, 6]. Lastly, there is a significant decrease in skin blood supply due to a reduced number of dermal blood vessels [7]. This is caused by endothelial dysfunction including reduced angiogenic capacity, aberrant expression of adhesion molecules, and impaired vasodilatory function [8]. The clinical signs include dermal atrophy and loss of elasticity [9].

Extrinsic aging is related to the long-term effects of ultraviolet (UV) radiation (sun) exposure and other environmental agents [10]. In contrast to the thinner epidermis in intrinsically aged skin, UV-radiated epidermis thickens, due to failed corneocyte desmosome degradation and an impaired epiderma keratinocyte differentiation process [11]. Photoaged skin shows decreased expression of type VII collagen in keratinocytes as well as an accumulation of abnormal elastic tissue deep in the dermis, a pathologic phenotype named solar elastosis [12]. Clinical signs include pigmentation changes, telangiectasias, corneal size increment, deep wrinkles, actinic keratosis, and precancerous and cancerous skin lesions.

Different therapeutic interventions have been suggested for skin aging including hormone replacement therapy, diets, anti-inflammatory drugs, reactive oxygen species (ROS) scavengers, and others [3]. However, most have shown limited success with little evidence [13].

Hyperbaric oxygen therapy (HBOT) utilizes 100% oxygen in an environmental pressure higher than one absolute atmosphere (ATA) to enhance the amount of oxygen dissolved in the body’s tissues. Repeated intermittent hyperoxic exposures have been shown to induce physiological effects which normally occur during hypoxia in a hyperoxic environment by the relative hypoxia exposure, the so called “hyperoxic-hypoxic paradox” [14–17]. These include mitochondrial function restoration and biogenesis, simulation of stem cell proliferation, migration, differentiation and angiogenesis among others [18]. Recent clinical trials have shown that HBOT can target aging hallmarks at the cellular level including telomere shortening and senescent cells clearance [19]. No study to date has examined HBOT’s effects on skin aging.

The aim of the current study was to evaluate the effects of HBOT on the skin of a normal, non-pathological, aging population.

## MATERIALS AND METHODS

### Subjects

Adults, living independently, in good functional and cognitive status without pathological cognitive decline, aged 64 and older, were enrolled. The study was performed between 2016-2020 in the Shamir (Assaf-Harofeh) Medical Center, Israel. Included patients did not have cardiac or cerebrovascular ischemia histories for the last year prior to inclusion. Exclusion criteria included: previous treatment with HBOT for any reason during the last three months, any history of malignancy during the last year, any pathological cognitive decline, severe chronic renal failure (GFR <30), uncontrolled diabetes mellitus (HbA1C>8, fasting glucose>200), taking immunosuppressants, MRI contraindications (including BMI>35), active smoking or pulmonary diseases.

### Study design

The study protocol was approved by the Institutional Review Board of the Shamir Medical Center, Israel. The study was performed as a prospective clinical trial. After signing an informed consent and undergoing a baseline evaluation, the subjects were assigned to a three-month control period followed by three months of HBOT daily sessions. Measurement points were evaluated at baseline, after three months of no intervention (control) and 1-2 weeks following the last HBOT session.

This study cohort, which is part of a larger cohort of normal ageing population studied at the Shamir Medical Center, Israel (NCT02790541), included only patients who consented to skin biopsies. The original design and sample size aimed to evaluate the primary endpoint related to cognitive function. Skin biopsies were a secondary endpoint and optional due to the invasiveness of the biopsy.

### Interventions

The HBOT protocol was administrated in a Multiplace Starmed-2700 chamber (HAUX, Germany). The protocol comprised of 60 daily sessions, five sessions per week within a three-month period. Each session included breathing 100% oxygen by mask at 2ATA for 90 minutes with five-minute air breaks every 20 minutes. Compression/decompression rates were 1 meter/minute. During both the control and HBOT periods, lifestyle and dietary changes, and medication adjustments were not allowed.

### Skin biopsies

Skin samples of 5X5 mm were taken from the post auricular regions by a 4mm punch biopsy technique after prepping, draping and local anesthesia. Biopsies were performed by a trained plastic surgeon. The chosen area was assumed to be protected from sun exposure, allowing the exclusion of the detrimental effects of photoageing or extrinsic skin ageing, and focusing on the intrinsic skin changes. Participants underwent skin biopsies at baseline, after the three-month control period and 1-2 weeks after the last HBOT session.

### Processing and staining

The samples were fixed in a 4% formaldehyde solution and underwent paraffinization. Histological sections of 5 micrometer width were performed with a microtome. Prior to staining, paraffin-embedded sections were deparaffinized and rehydrated. Processing and staining were performed by a blinded trained pathology technician. All slides were imaged using a Lionheart™ FX microscope. Sections were examined and evaluated in random order under blinded conditions with light or fluorescent microscopy. Samples with low quality specific staining were not quantified. Importantly, slides were evaluated in a blinded manner, and missing parameters were filled in as blanks rather than numbers.

### Collagen fibers staining

Trichrome stain was performed using Abcams' kit (ab150686, ABCAM). Briefly, the sections were incubated in Bouins fluids for one hour at 56-64° C degrees and then washed with water. Next, the sections were transferred to Weigert's iron solution for five minutes followed by a rinse with water and then transferred to acid fuchsin solution for 15 minutes. Then, sections were rinsed with distilled water and incubated for 15 min in phosphomolybdic acid solution followed by Aniline blue for 10 minutes and 1% acetic acid for 5 minutes. Following completion, sections were dehydrated, cleared in xylene and mounted with Entellan.

### Epidermis layer thickness

Thickness of the epidermis was calculate as micrometers (μm) digitally (X200) using Gene5 software, image analyzing system (BioTek). The epidermis thickness was measured five times from the free margin of skin to the dermal papillae and epidermal rete ridge.

### Papillary layer thickness

As described by Marcos-Garces et al. [6], 5 representative measurements of the papillary dermis thickness were selected using the X100 magnification. This was possible due to the contrast produced between the thick collagen bundles integrating the reticular dermis and the thin collagen bundles forming the papillary dermis.

### Papillary layer collagen fibers thickness

As described by Marcos-Garces et al. [6], the thickness of 15 collagen bundles was measured using the X400 magnification photographs, enlarging the image so that the limits of the bundles could be properly discerned.

### Area occupied by collagen in the reticular layer

To determine the density of the collagen bundles within the reticular dermis, we selected the area occupied by collagen in the X400 magnification of the reticular dermis using a semi-automated procedure. In the images, the area corresponding to the reticular dermis was manually selected and the density was calculated using the Gene5 software confluence tool.

### Reticular layer collagen fibers thickness

The thickness of 15 collagen bundles was measured using the X200 magnification micrographs. The image was manually enlarged till distinct visualization of the limits between bundles were seen, as described by Marcos-Garces et al. [6].

### Elastic fibers staining

Orcein stain for elastic fibers was performed using the designated kit (1.07100.0025, SIGMA). Briefly, sections were incubated in Orcein solution for 30 minutes, followed by hematoxylin solution for 30 seconds. The sections were then dehydrated, cleared with Xylene and mounted with Entellan.

### Reticular layer elastic fibers thickness and length

The thickness and length of 15 elastic bundles were measured using X400 magnification micrographs. The image was manually enlarged till distinct visualization of the limits between bundles were seen.

### Area occupied by elastin in the reticular dermis

Semi-quantitative estimation of histochemical staining was carried out independently by two blind investigators. Each investigator analyzed five areas per specimen under light microscopy using X400 magnification.

Elastin fibers density (%) within the reticular layer was evaluated in each of the five areas and the mean was calculated. Elastin fragmentation scored from 1-3, with 3 representing high fragmentation and 1 no fragmentation.

### Tissue senescent cells count

Senescence evaluation was performed using Sudan Black B staining (SBB) as previously described by Georgakopoulou et al. [20]. Briefly, freshly prepared SBB was dropped on a clean slide. The dehydrated tissue was placed face down on the drop of SBB on the slide and incubated for 5 minutes. The slide was carefully lifted and the SBB was manually wiped from the back and along the slide using an absorbent soft paper. The tissues were then embedded in 50% ethanol and washed in distilled water. Next, they were counterstained with 0.1% nuclear fast red (NFR) (SIGMA) for 10 min and mounted using 40% Glycerol/Tris Buffered Saline (TBS) mounting medium.

Sections were examined and evaluated in random order under blinded conditions with light microscopy. Senescence assessment was done by lipofuscin expression with a score from 1-5, with 1 being negative staining for lipofuscin and 5 high positive staining.

### Tissue blood vessels count

Blood vessels evaluation was carried out as follows: tissue underwent antigen retrieval using 1 mM EDTA supplemented with 0.05% Tween 20, pH 8.0 for 40 min at 95-100° C. After blocking with 5% normal horse serum for 1 hour at RT, sections were incubated with anti-CD31 primary antibody (R&D Systems 1:40) overnight at 4° C, followed by donkey anti-goat secondary antibody (ABCAM 1:200) for one hour at RT. Sections were then mounted with Fluorosheild mounting medium with DAPI (ab104139, ABCAM). Control samples were exposed only to the secondary antibody to rule out non-specific staining.

Stained slides were imaged for fluorescence using the Lionheart™ FX Automated Fluorescent Microscope. Three random fields at X20 magnification were captured for each section. The blood vessels were counted and the mean fluorescence intensity (MFI) was measured. Furthermore, total surface area covered by all the blood vessels was calculated by averaging the two diameters of each vessel and multiplying it by the total number of blood vessels in the same ROI.

### Statistical analysis

Unless otherwise specified, continuous data were expressed as means ± standard-deviation (SD). The normal distribution for all variables was tested using the Kolmogorov-Smirnov test. One-way ANOVA was performed to compare variables between and within the three groups at baseline.

Categorical data is expressed in numbers and percentages and compared by chi-square tests. Univariate analyses were performed using chi-square/Fisher’s exact test to identify significant variables (P<0.05).

To evaluate HBOT’s effects, a repeated measures ANOVA model was used to test the main within-subject effect. Post-hoc tests on the means were conducted to test for time differences using paired t tests with a Bonferroni correction.

Net effect size was calculated by the subtraction of Cohen’s D effect size of the control period (control-baseline) from the HBOT period (HBOT-control) Cohen’s D effect size.

## RESULTS

Out of the cohort of 70 participants in the normal ageing population study, thirteen patients gave consent for skin biopsies. All thirteen patients completed three biopsies (baseline, three-month control period and post-HBOT). Only males gave consent and their mean age was 68.07±2.5y. The baseline characteristics of the patients are summarized in Table 1.

**Table 1:** Baseline characteristics.

	**HBOT**
**N**	13
**Age (years)**	68.07±2.5
**Males**	13 (100%)
**Chronic medical conditions**	
Skin basal cell carcinoma	3 (23.0%)
Atrial fibrillation	0
Hypothyroidism	0
Obstructive sleep apnea	0
Asthma	1 (7.6%)
BPH	2 (15.3%)
GERD	1 (7.6%)
Osteoporosis	0
Rheumatic arthritis	0
Osteoarthritis	1 (7.6%)
Diabetes mellitus	2 (15.3%)
Hypertension	4 (30.7%)
Dyslipidemia	8 (61.5%)
Ischemic heart disease	4 (30.7%)
History of smoking	6 (46.1%)
**Chronic medications**	
Anti-aggregation	5 (38.4%)
ACE-Inhibitors/ARB blockers	5 (38.4%)
Beta blockers	4 (30.7%)
Calcium blockers	3 (23.0%)
Alpha blockers	3 (23.0%)
Diuretics	0
Statins	8 (61.5%)
Oral hypoglycemic	2 (15.3%)
Bisphosphonates	0
Proton pump inhibitors	2 (15.3%)
Hormones	0
Benzodiazepines	2 (15.3%)
SSRI	2 (15.3%)

### Elastic fibers

**Figure 1:**
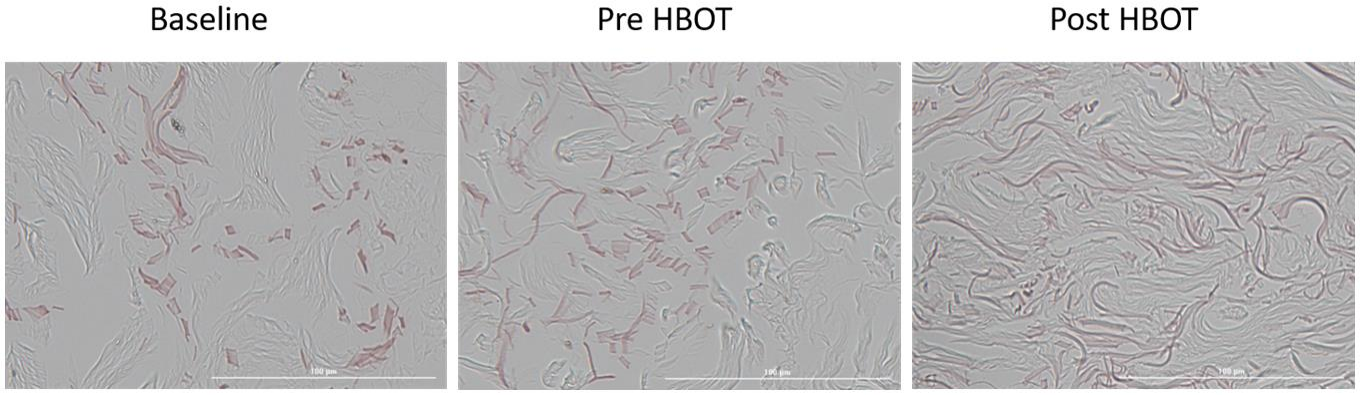
**Elastic fiber changes.** Following HBOT, there was a significant increase in elastic fiber numbers, along with a significant decrease in their fragmentation.

Elastic fibers were evaluated using Orecin staining. There were no significant changes in the elastic fibers’ length following the control period (6.94±1.45 vs. 5.83±1.59, p=0.091). Following HBOT, there was a significant increase in the fibers’ length to 14.25±4.31 (p<0.0001, effect size=2.71, Table 1 and Figure 1). Additionally, there was a significant decrease in the fibers’ fragmentation following HBOT (90% vs 10% high fragmentation, p=0.012, Table 1). No changes were noted in the elastic fibers’ density or thickness (Table 2).

**Table 2:** Elastic and collagen fibers changes.

	**Baseline**	**Control**	**Post HBOT**	**Repeated measures Sig.**	**Baseline - control Sig.**	**Control - HBOT Sig.**	**Baseline - HBOT Sig.**	**Net effect size**
**Elastic fibers**								
**Length (μm) (N=11)**	6.94±1.45	5.83±1.59	14.25±4.31	**<0.0001**	0.091	**<0.0001**	**0.001**	2.71[0.46-4.96]
**Thickness (μm) (N=11)**	2.51±0.99	2.44±0.66	2.20±0.24	0.48	0.812	0.286	0.369	0.23[(-0.76)-1.23]
**Density (%) (N=11)**	80.5±11.89	75.5±19.21	78.5±11.31	--	0.863 (W)	0.952 (W)	0.619 (W)	0.45[(-0.70)-1.61]
**Fragmentation (N=11)**				**0.012**				
**Low**	0	0	3 (30%)					
**Medium**	1 (10%)	4 (40%)	5 (50%)					
**High**	9 (90%)	6 (60%)	2 (10%)					
**Collagen fibers**								
**Density (%) (N=12)**	67.8±10.9	67.9±8.6	76.6±23.15	**0.001**	0.984	**<0.001**	**0.014**	1.10[(-0.04)-2.25]
**Fibers thickness in Papillary layer (μm) (N=11)**	2.81±0.83	2.63±0.42	3.02±0.79	0.421	0.570	0.156	0.515	0.61[(-0.56)-1.78]
**Fibers thickness in the reticular layer (μm) (N=11)**	5.21±1.19	4.98±1.40	5.95±1.52	0.261	0.730	0.100	0.258	0.61[(-0.55)-1.78]
**Papillary layer thickness (μm) (N=11)**	133.22±22.42	125.64±35.18	106.60±31.01	**0.007**	0.395	**0.009**	**0.004**	0.47[(-0.70)-1.65]
**Epidermis thickness (μm) (N=11)**	55.88±9.19	58.21±12.32	57.56±14.20	0.838	0.645	0.871	0.684	0.20[(-0.93)-1.34]

### Collagen fibers

**Figure 2:**
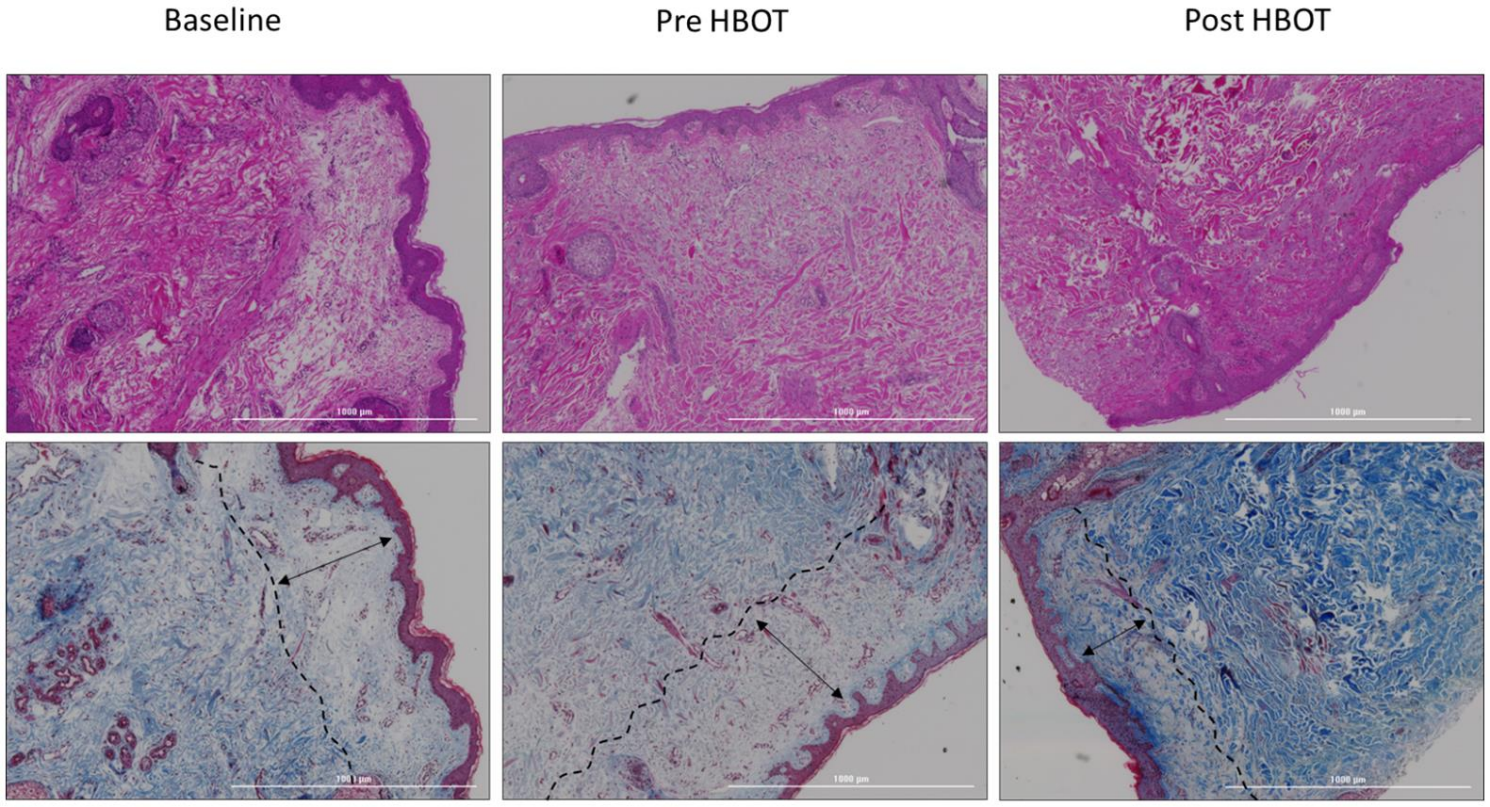
**Papillary layer thickness changes.** Following HBOT, there was a significant decrease in the papillary layer thickness.

The collagen fibers were evaluated using Trichrome staining. There were no changes in the collagen fibers density following the control (67.84±10.98 vs. 67.90±8.62, p=0.984) with a significant increase demonstrated following HBOT (76.61±9.52, p<0.0001, effect size=1.10). In addition, there were no changes in the papillary layer thickness following the control period (133.22±22.42 vs. 125.64±35.18, p=0.395), whereas a significant decrease was noted following HBOT (106.60±31.01, p=0.009, effect size=0.61) (Table 2 and Figure 2). No significant changes in the collagen fiber thickness in both papillary and reticular layers were demonstrated (Table 2). the epidermis layer thickness did not change as well (Table 2).

### Blood vessels

**Figure 3:**
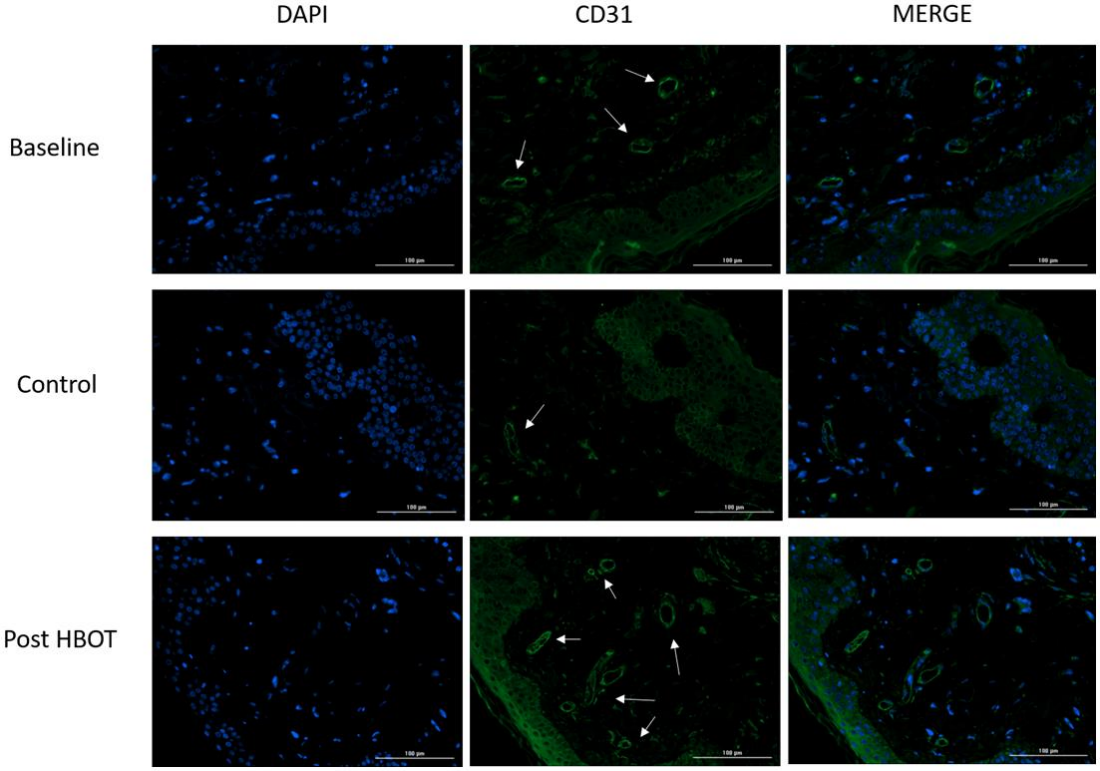
**Blood vessels changes.** Following HBOT, there was a significant increase in tissue blood vessels. Left column: DAPI staining for intact cells evaluation; middle column: CD31 staining of endothelial cells; right column: merging of DAPI and CD31.

Endothelial cells were evaluated using CD31 staining. No significant changes were noted in either CD31 intensity staining (393.63±167.83 vs. 339.45±301.63, p=0.521) or vessels count (24.00±15.72 vs. 23.70±10.28, p=0.943) following the control period (Table 3). There was a significant increase in CD31 intensity (625.81±394.29, p=0.04, effect size=1.00) and the number of vessels (33.40±12.28, p=0.02, effect size=0.84) post-HBOT (Table 3 and Figure 3).

**Table 3:** Blood vessels and senescence cells changes.

	**Baseline**	**Control**	**Post HBOT**	**Repeated measures Sig.**	**Baseline - control Sig.**	**Control - HBOT Sig.**	**Baseline - HBOT Sig.**	**Net effect size**
**Blood Vessels (N=10)**								
**CD31 intensity**	393.63±167.83	339.45±301.63	625.81±394.29	**0.044**	0.521	**0.040**	0.074	1.00[(-0.28)-2.29]
**Count**	24.00±15.72	23.70±10.28	33.40±12.28	**0.039**	0.943	**0.020**	**0.049**	0.84[(-0.46)-2.13)
**Senescence (N=11)**	3.14±1.06	3.13±1.10	2.48±1.23	**0.016**	0.956	**0.033**	**0.012**	0.90[(-0.42)-2.22]

### Senescence cells

**Figure 4:**
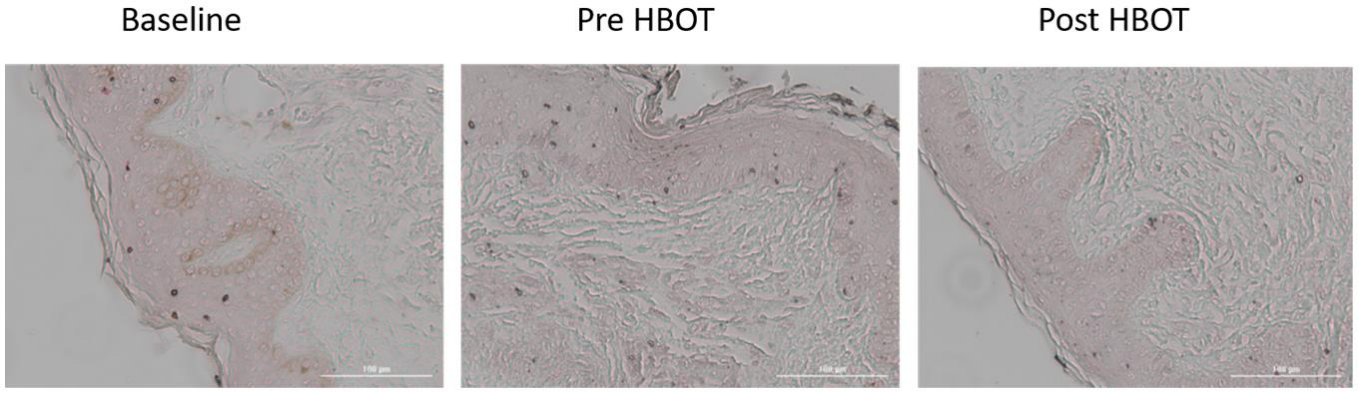
**Senescence cells changes.** Following HBOT, there was a significant decrease in number of tissue senescent cells.

Tissue senescent cells were evaluated using the lipofuscin staining method. No changes were noted in senescent cells following the control period (3.14±1.06 vs. 3.13±1.10, p=0.956) (Table 3). There was a significant decrease in tissue senescent cells (2.48±1.23, p=0.033) (Table 3 and Figure 4) post-HBOT.

### Safety

Out of the 13 participants, two side effects were reported. One patient had a local infection at the biopsy site which was treated with a three-day oral antibiotics course. Another patient suffered from moderate barotrauma to the ear which was treated conservatively with complete resolution.

## DISCUSSION

The current study demonstrates, for the first time, that 60 daily HBOT sessions decreased papillary dermis thickness, increased elastic fiber length and stability and collagen fibers density with a large effect size in a healthy elderly cohort. Moreover, HBOT beneficially impacted on both the number of blood vessels and senescent cells. This is the first human study that demonstrates that a therapeutic intervention can reduce the number of senescent cells at the tissue level.

Skin aging begins from birth and is induced by both intrinsic and extrinsic factors. We focused on intrinsic aging by obtaining skin biopsies from a light protected area. The intrinsic physiological aging includes the so called classical aging process which includes, increased senescent cells (fibroblasts, melanocytes) and loss/degradation of the microcirculation, in addition to epidermis thinning, dermis thinning with increased papillary layer thickness, decreased collagen deposition, elastic fibers shortening and degradation [3]. Thus, this accessible skin biopsy method enabled us to gain better understanding of HBOT’s effects at the tissue level. As shown in this study, HBOT had significant aging modulation effects by decreasing senescent cells, inducing angiogenesis and increasing elastic fiber length and stability and collagen density.

Different models have been proposed to explain the molecular basis for skin aging based on the hallmarks of aging including cellular senescence, loss of telomeres, DNA mutations, mitochondrial dysfunction, oxidative stress, chronic inflammation and others [21]. In the past decade, both preclinical and clinical studies demonstrated HBOT can target many of those aging hallmarks. HBOT can improve mitochondrial function and cellular metabolism, reduce inflammatory reactions, reduce apoptosis, alleviate oxidative stress and aid in the initiation or facilitation of angiogenesis [22]. Although HBOT might be expected to induce significant oxidative stress, it has been shown that only a single hyperoxic exposure increases ROS generation acutely, whereas repeated exposures trigger an anti-oxidant/scavenger response, becoming protective with normalized ROS levels [18].

In the current study we focused on two of HBOT’s mechanisms: angiogenesis and reduced cell senescence. Decreased tissue perfusion, or bloody supply has been associated with skin aging [7]. In our study, HBOT induced angiogenesis, with increased dermal blood vessels. The results correlate with our previous studies demonstrating that HBOT can induce angiogenesis in ischemic non-healing wounds and in the aging brain [23–25]. Our HBOT protocol utilizes the effects induced by repeated intermittent hyperoxic exposures, the so called hyperoxic hypoxic paradox [14–17]. These intermittent hyperoxic exposures induce physiological responses that occur during hypoxia. Thus, HBOT induces the release of the transcription factors called hypoxic induced factors (HIFs) and increases their stability and activity. In turn, HIF-1α and HIF-2α modulate the release of the angiogenic factor vascular endothelial growth factor (VEGF). VEGF is considered the master regulator of angiogenesis, and induces migration of progenitor endothelial cells from the bone marrow into the circulatory system, recruitment of endothelial cells from existing blood vessels, and their differentiation into newly formed blood vessels [14–17]. HBOT induced angiogenesis may play a significant impact on photoaging and extrinsic skin aging where blood vessels are more severely impaired due to UV and toxic exposures. However, the current study did not evaluate light exposed skin.

Second, intrinsic skin aging is associated with cell senescence, where the cell-cycle of fibroblasts, melanocytes and others cells are arrested. The main driving forces for senescence include telomere shortening [26], as well other non-telomeric DNA damage [27]. The accumulation of senescent cells with aging reflects either an increase in the generation of these cells and/or a decrease in their clearance, which in turn aggravates the damage and contributes to aging [27]. In the current study, HBOT induced a significant decrease/clearance in cell senescence compared to the stable levels of cell senescence at baseline and following a control period. In our previous work, we demonstrated that HBOT induces peripheral blood senescent cell clearance along with telomere elongation [19]. Both of these support similar effects on tissue senescent cells, such as the skin.

HBOT has been utilized for decades as a therapeutic modality for non-healing wounds, compromised skin grafts and flaps, severe skin infections, necrotizing soft tissue infections, burns, crush syndrome and other acute ischemia injuries [28]. Most recently, it has been suggested as preconditioning prior to aesthetic surgeries [29]. However, to the best of our knowledge, this is the first study evaluating its effects on the typical aging skin.

Currently, many interventions that genetically or pharmacologically (senolytic drugs) target skin aging hallmarks are being investigated. These include antioxidants, stem cell therapy, retinoids, hormones, diets, anti-inflammatory agents, anti-progeria strategies, telomere modifications and others [3]. Most of these interventions have scant evidence backing their claims with limited effects [3]. Moreover, most of the evidence is from animal models and awaits safety and efficacy evaluations in humans. Notably, topical retinoids have shown increased epidermis thickness [3]. The current study suggests a non-pharmacological method for senescent cells removal. For the first time in humans, a therapeutic intervention demonstrated an actual reduction in in the number of senescent cells at the tissue level.

Study limitations: The current study has several limitations and strengths to consider. First, the limited sample size should be considered. Second, there was a lack of a separate placebo group. However, the patients did perform a second biopsy after a control period for comparison and the biopsy analysis was performed in a blinded fashion. Third, the skin was not evaluated and scored clinically. Fourth, HBOT effects on photoaging were not evaluated. Fifth, we evaluated two HBOT related mechanisms, angiogenesis and senescent cells clearance, however other skin aging reverting mechanisms can be in play and were not evaluated. Lastly, our protocol included 60 sessions of 100% oxygen at 2 ATA including three air breaks during each session to take advantage of the hyperoxic hypoxic paradox and minimize the risk for oxygen toxicity. We suggest both within session and between sessions hyperoxic-hypoxic / relative hypoxic exposures affect the discussed cascade; However, the current experimental design cannot evaluate each component separately. Additionally, the dose response curve related to the applied pressure, time and number of HBOT exposures and its relation to HIF expression and its related reverse aging effects are still not fully understood and further studies are needed to find the optimal HBOT protocols.

In summary, for the first time in humans, our study indicates that HBOT can significant modulate pathophysiological aging effects on the skin of healthy aging adults. The demonstrated mechanisms include angiogenesis and clearance of senescent cells.
